# Supplementary material for: Spatial multi-omics of human skin reveals KRAS and inflammatory responses to spaceflight
Source: Nat Commun. 2024 Jun 11;15:4773. doi: 10.1038/s41467-024-48625-2 (PMC11166909; doi:10.1038/s41467-024-48625-2)
Supplement: Supplementary file 1 — Supplementary Information [file 41467_2024_48625_MOESM1_ESM.pdf]

SUPPLEMENTARY FIGURES

Supplementary Fig.1

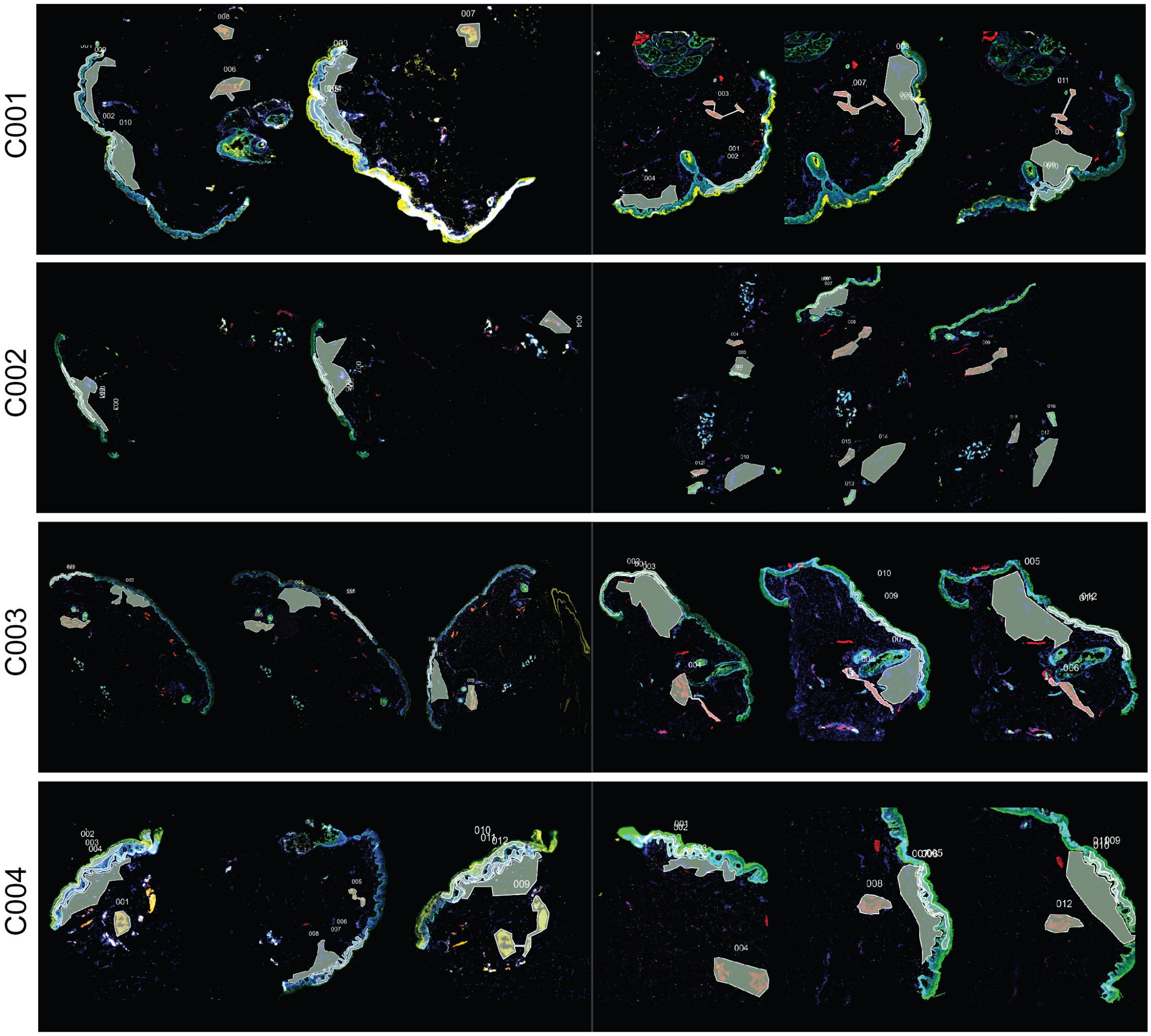

Supplementary Fig. 1. **GeoMx ROI selection.** Stained images and ROI selections for all GeoMx samples

Supplementary Fig.2

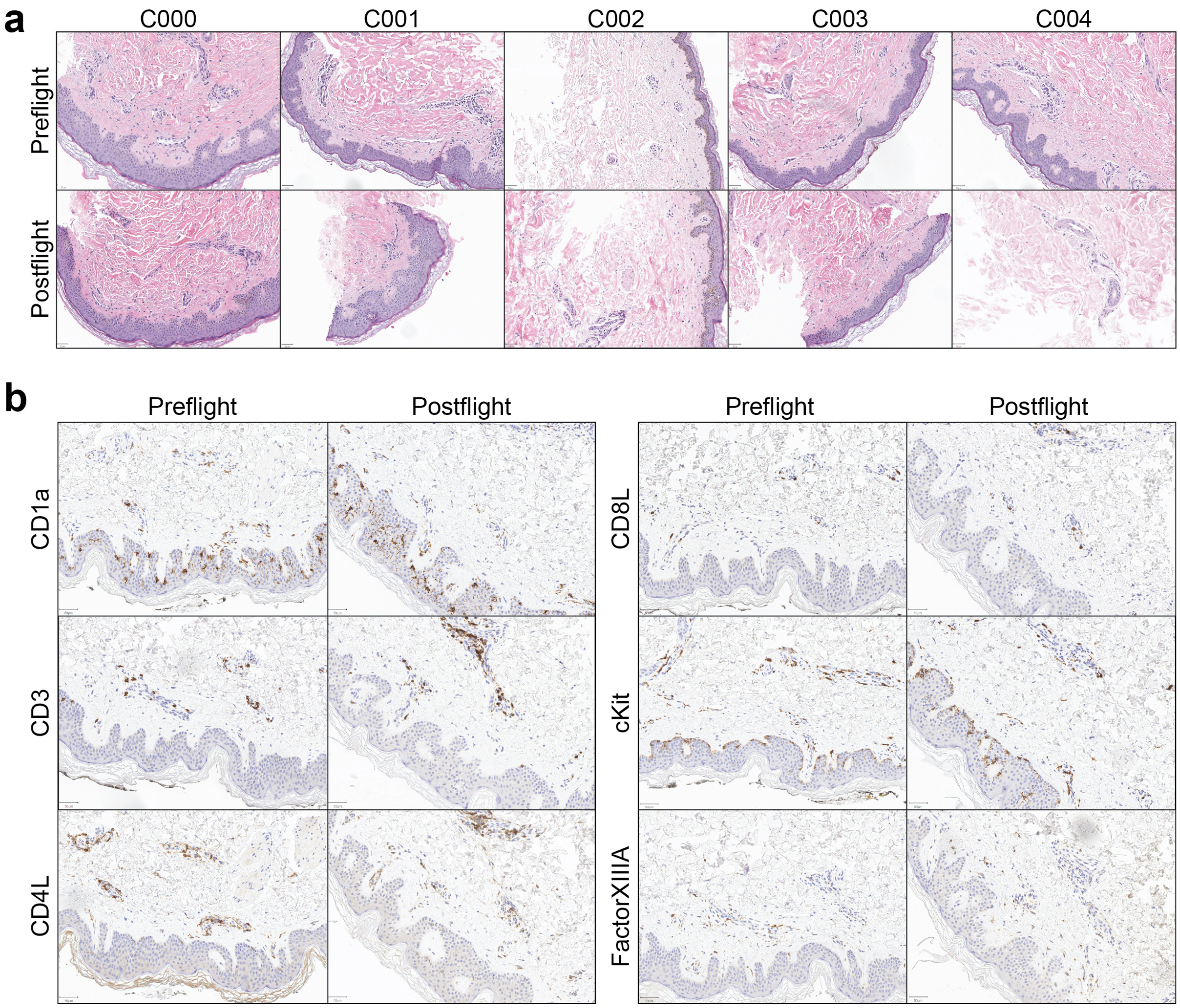

Supplementary Fig. 2. Representative tissue images. (a) H&E, and (b) IHC staining images of the crews' skin biopsy slides; C000 is ground control and scale bar size 50 µm.

Supplementary Fig.3

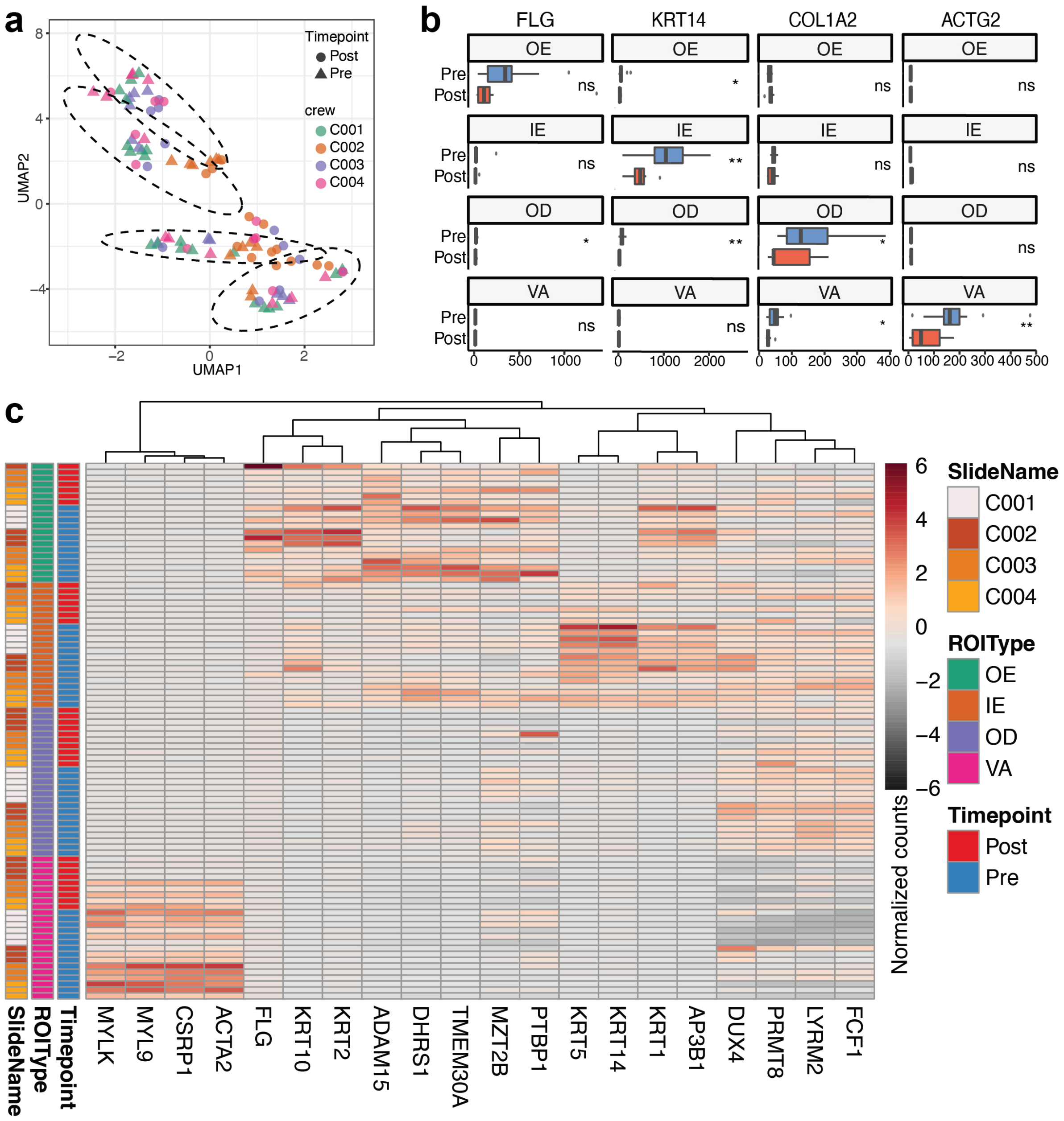

Supplementary Fig. 3. **GeoMx data analysis.** (a) UMAP visualization by crew members, where dotted line indicates ROI type labels, (b) Skin protein associated gene expression levels (p-values were determined by two-sided Wilcoxon test across 95 ROIs and four crews; ns, non-significant, $*p \leq 0.05$, $**p \leq 0.01$, $***p \leq 0.001$, and $****p \leq 0.0001$) and (c) Expression levels of top 20 ROI type variable genes across all ROIs.

Supplementary Fig.4

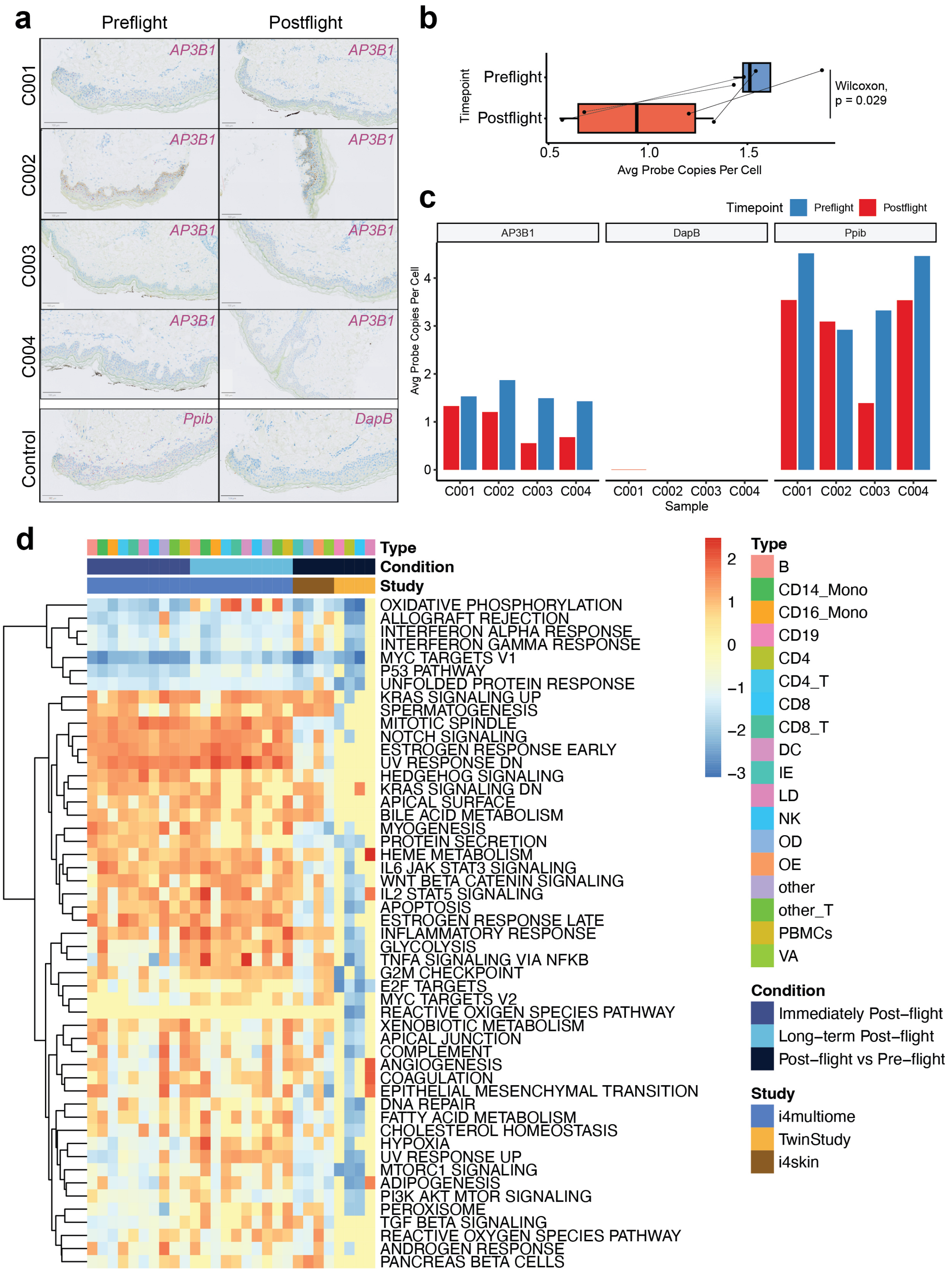

Supplementary Fig. 4. **Data validation and comparison.** (a) RNA scope images to quantify *AP3B1* transcripts in the preflight and postflight tissue biopsies, (b) Boxplot visualizing average probe copies per cell within the epidermis region (p-value was determined by two-sided Wilcoxon test, and boxplot shows median/horizontal line inside the box, the interquartile range/box boundaries, whiskers extending to 1.5 times the interquartile range, and outliers as individual points outside the whiskers), (c) Average probe copies identified per cell in the epidermis region, where DapB is negative control probe and Ppib is positive control probe, and (d) Pathway-level comparisons with previous twin study human spaceflight data (using fgsea method). The colors of the heatmap indicate normalized enrichment (NES) value.

Supplementary Fig.5

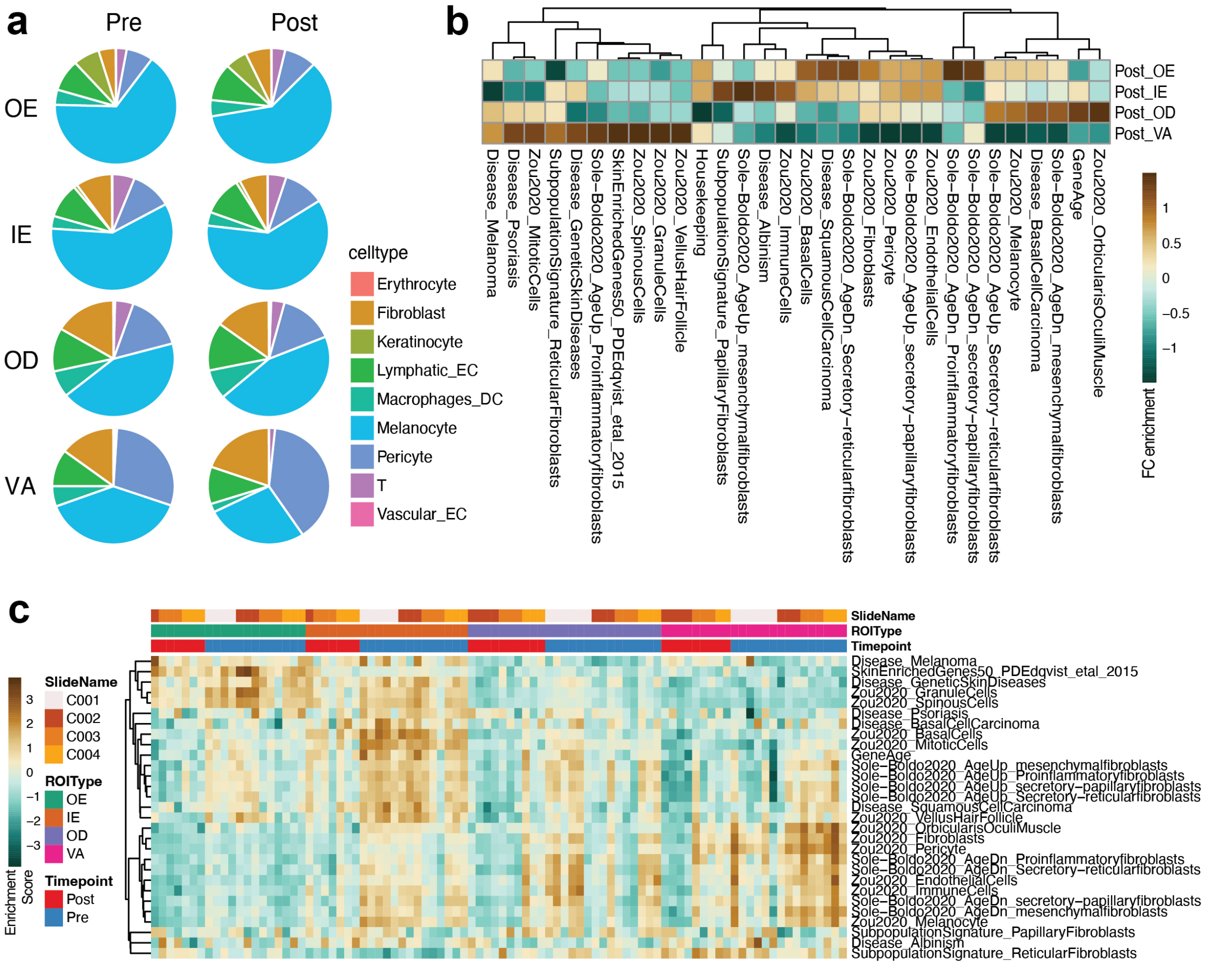

Supplementary Fig. 5. **Geneset enrichment analysis.** (a) broad cell proportion changes from GeoMx data by timepoint and ROI types, and ssGSEA results from published skin datasets visualizing (b) fold change enrichment each ROI type, and (c) scaled enrichment scores across all ROIs.

Supplementary Fig.6

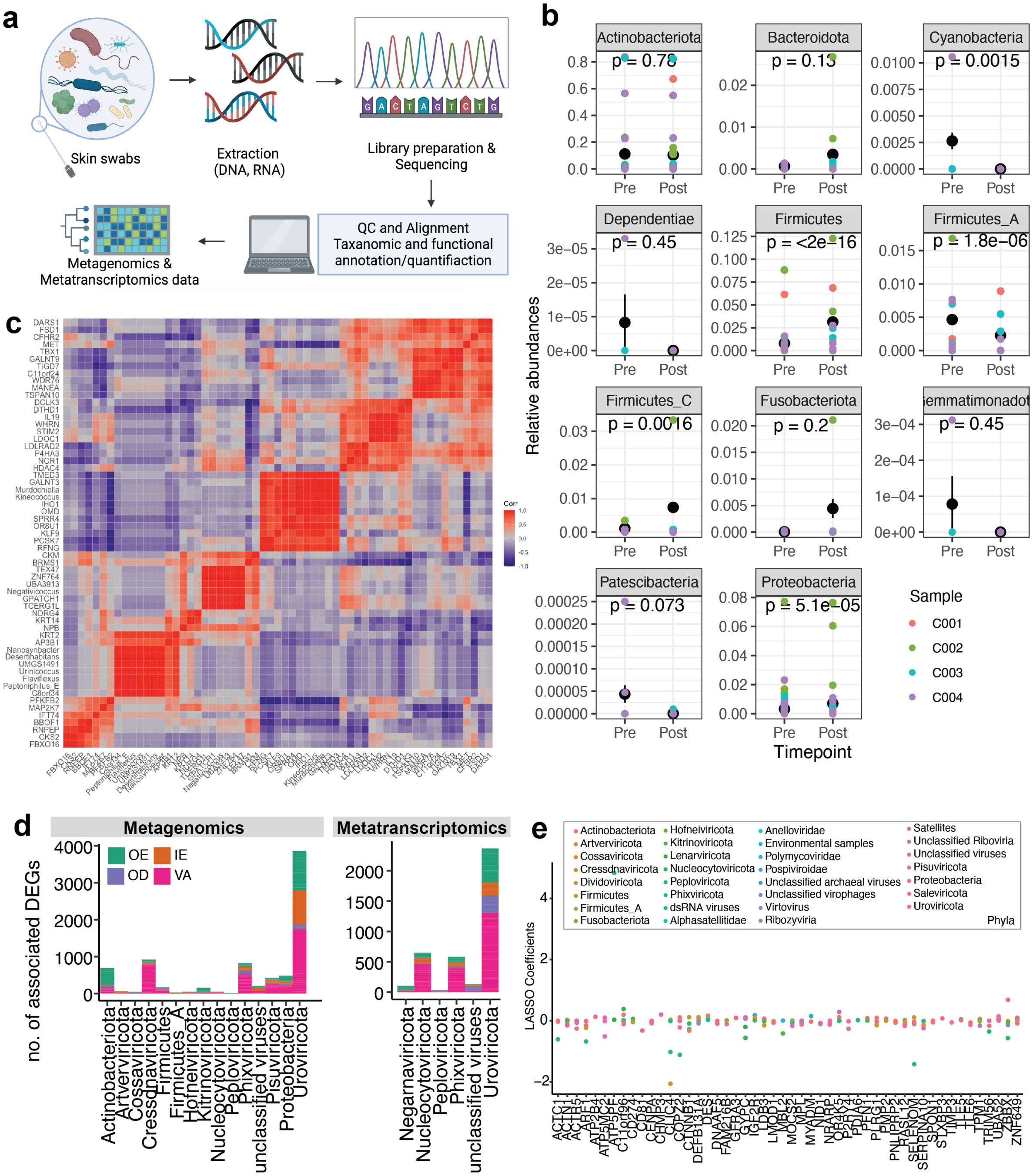

Supplementary Fig. 6. Integration with metagenomics/metatranscriptomics data. (a) Metagenomics and transcriptomics data processing workflow, and (b) Relative abundance metagenomic changes and statistics by phyla, p-values were calculated by two-sided Wilcoxon test, (c) Pearson correlation between top 25 bacterial species of differential abundance change (effect size) and 25 OE DEGs, and (d) number of associated human genes with phyla from metagenomics (left) and metatranscriptomics (right) data, and (e) LASSO coefficients to probe strength of the association between phyla and human genes identified by spatial transcriptomics in outer epidermal (OE) region.

Supplementary Fig.7

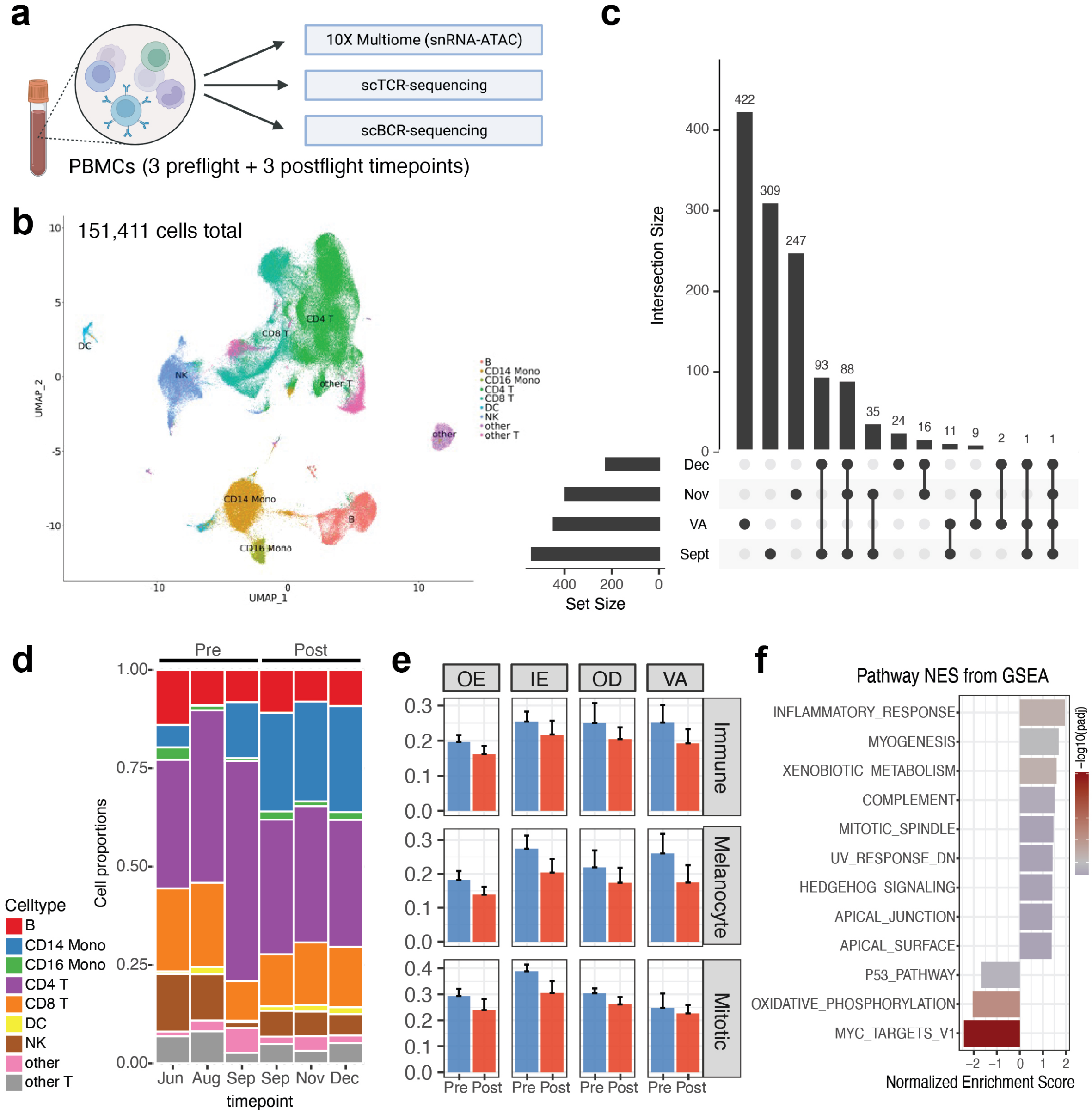

Supplementary Fig. 7. Integration with immune single-cell data. (a) Experimental design for PBMCs, (b) UMAP projection of all cells with identified cell types, and (c) UpSetR Plot comparing overlapping DEGs (relative to preflight timepoints) across all postflight timepoints; September postflight timepoints are collected on the same day as spatial postflight biopsies, and two additional postflight timepoints were taken in November and December, (d) cell proportion changes across timepoints within PBMCs, (e) cell signature changes from ssGSEA analysis of the skin spatial data by regions (across 95 ROIs total, error bars indicate standard deviation), and (f) Pathway enrichment analysis (using MSigDB Hallmark pathways) results from PBMC multiome dataset, visualized pathways that makes the p-value cutoff < 0.1 using fgsea method (KS test).
