# Supplementary material for: Spatial multi-omics of human skin reveals KRAS and inflammatory responses to spaceflight
Source: Nat Commun. 2024 Jun 11;15:4773. doi: 10.1038/s41467-024-48625-2 (PMC11166909; doi:10.1038/s41467-024-48625-2)
Supplement: Supplementary file 3 — Description of Additional Supplementary Files [file 41467_2024_48625_MOESM3_ESM.pdf]

DESCRIPTION OF ADDITIONAL SUPPLEMENTARY FILES

Supplementary Data 1. Differential expression analysis results and statistics

Supplementary Data 2. Gene set enrichment analysis results and statistics

Supplementary Data 3. Relative abundances of bacterial and viral species from skin swab metagenomics and metatranscriptomics analysis and LASSO association analysis results
