# Supplementary material for: Spatial multi-omics of human skin reveals KRAS and inflammatory responses to spaceflight
Source: Nat Commun. 2024 Jun 11;15:4773. doi: 10.1038/s41467-024-48625-2 (PMC11166909; doi:10.1038/s41467-024-48625-2)
Supplement: Supplementary file 7 — Reporting Summary [file 41467_2024_48625_MOESM7_ESM.pdf]

nature portfolio

Corresponding author(s): Christopher E. Mason

Last updated by author(s): Oct 1, 2023

Reporting Summary

Nature Portfolio wishes to improve the reproducibility of the work that we publish. This form provides structure for consistency and transparency in reporting. For further information on Nature Portfolio policies, see our Editorial Policies and the Editorial Policy Checklist.

Statistics

For all statistical analyses, confirm that the following items are present in the figure legend, table legend, main text, or Methods section.

n/a	Confirmed	
☐	☒	The exact sample size (*n*) for each experimental group/condition, given as a discrete number and unit of measurement
☐	☒	A statement on whether measurements were taken from distinct samples or whether the same sample was measured repeatedly
☐	☒	The statistical test(s) used AND whether they are one- or two-sided<br>*Only common tests should be described solely by name; describe more complex techniques in the Methods section.*
☐	☒	A description of all covariates tested
☐	☒	A description of any assumptions or corrections, such as tests of normality and adjustment for multiple comparisons
☐	☒	A full description of the statistical parameters including central tendency (e.g. means) or other basic estimates (e.g. regression coefficient) AND variation (e.g. standard deviation) or associated estimates of uncertainty (e.g. confidence intervals)
☐	☒	For null hypothesis testing, the test statistic (e.g. *F*, *t*, *r*) with confidence intervals, effect sizes, degrees of freedom and *P* value noted<br>*Give P values as exact values whenever suitable.*
☒	☐	For Bayesian analysis, information on the choice of priors and Markov chain Monte Carlo settings
☒	☐	For hierarchical and complex designs, identification of the appropriate level for tests and full reporting of outcomes
☒	☐	Estimates of effect sizes (e.g. Cohen's *d*, Pearson's *r*), indicating how they were calculated

Our web collection on statistics for biologists contains articles on many of the points above.

Software and code

Policy information about availability of computer code

Data collection	Collected data was processed with following pipelines:<br>1. GeoMx NGS Pipeline (DND) Processing Nanostring GeoMx NGS data for WTA and CTA assays, version 1.0.0; https://blog.nanostring.com/geomx-online-user-manual/Content/NGS_DND/Running_DND.htm#Running3<br>2. 10X Cell Ranger pipeline: https://support.10xgenomics.com/single-cell-gene-expression/software/pipelines/latest/using/tutorial_ov
Data analysis	Codes used to analyze skin spatial transcriptomics, processed data used to generated figures, and skin-specific custom gene sets are accessible in GitHub repository: https://github.com/jpark-lab/SpatialAnalysis/. All other data analyses including single cell multiome and metagenomics data analysis can be found in https://github.com/eliah-o/inspiration4-omics. Additional information and interactive online visualization tools are accessible at: https://epigenetics.weill.cornell.edu/apps/SOMA_Browser/<br><br>The following packages were used and cited in the manuscript:<br>1. DESeq2, R package for analysis differential gene expression,version 1.28.0 for bulk RNA seq analysis, version 1.30.0 for viral and spatial DE analysis http:/ /bioconductor.org/packages/release/bioc/htm l/DESeq2. html<br>2. MuSiC, R package for estimation of cell type proportions in bulk RNA-seq data, version 0.1.1; https://github.com/xuranw/MuSiC<br>3. GSEA, software for analyzing gene set enrichments, version 4.1.0 (run with database available as of 11/1/2020); https://www.gsea-msigdb.org/gsea/index.jsp<br>4. Seurat, software for analyzing single-cell level datasets; version 4.4.0 https://github.com/satijalab/seurat<br>5. Limma,R package for differential gene expression analysis for NanoString GeoMx data, version 3.44.3;http://bioconductor.org/packages/release/bioc/html/limma.html<br>6. edgeR, R package for differential gene expression analysis for NanoString GeoMx data, version 3.30.3 ;https://bioconductor.org/packages/release/bioc/html/edgeR.html

7. EnhancedVolcano, R package for generating volcano plots for differential genes for analysis on NanoString GeoMx data, versionl.6.0; https ://bioconductor .org/packages/release/bioc/htm I/En hancedVolcano. htm I
8. fgsea, R package for gene enrichment analysis on NanoString GeoMx data, version 1.14.0; http://bioconductor.org/packages/release/bioc/html/fgsea.html
9. Venn, website for generating Venn diagrams; http://bioinformatics.psb.ugent.be/webtools/Venn/
10. Cocor, R package for Comparing Correlations, version 1.1-3, https://rdrr.io/cran/cocor/
11. synRNASeqNet, Synthetic RNA-Seq Network Generation and Mutual Information Estimates, https://cran.r-project.org/web/packages/synRNASeqNet/index.html
12. Nf-core/rnaseq, bioinformatics analysis pipeline used for RNA sequencing data, https://github.com/nf-core/rnaseq
13. MiXCR, software for fast and accurate analysis of raw T- or B- cell receptor repertoire sequencing data, https://github.com/milaboratory/mixcr
14. GSVA, Gene Set Variation Analysis for microarray and RNA-seq data, https://www.bioconductor.org/packages/release/bioc/html/GSVA.html
15. Pyrpipe, python package for RNA-Seq workflows, https://github.com/urmi-21/pyrpipe
16. MetaOmGraph, a workbench for interactive exploratory data analysis of large expression datasets, https://github.com/urmi-21/MetaOmGraph
17. IsoformAnalyzeR, an R package to detect isoform switching from RNA-seq data. Version 1.8.0. https://www.bioconductor.org/packages/release/bioc/html/IsoformSwitchAnalyzeR.html
18. PathwaySplice, an R package to detect pathway enrichment with awareness of the number of features per gene. Version 1.5.0. https://www.bioconductor.org/packages/release/bioc/html/IsoformSwitchAnalyzeR.html

For manuscripts utilizing custom algorithms or software that are central to the research but not yet described in published literature, software must be made available to editors and reviewers. We strongly encourage code deposition in a community repository (e.g. GitHub). See the Nature Portfolio guidelines for submitting code & software for further information.

Data

Policy information about availability of data

All manuscripts must include a data availability statement. This statement should provide the following information, where applicable:

- Accession codes, unique identifiers, or web links for publicly available datasets
- A description of any restrictions on data availability
- For clinical datasets or third party data, please ensure that the statement adheres to our policy

All the raw sequencing data (skin spatial transcriptomics, swab metagenomics and metatranscriptomics) as well as images, processed data, and associated metadata are submitted to NASA GeneLab data sharing platform (https://osdr.nasa.gov/bio/repo/data/studies/OSD-574). PBMC single-cell multiome data is also available via NASA GeneLab platform (https://osdr.nasa.gov/bio/repo/data/studies/OSD-570).

Research involving human participants, their data, or biological material

Policy information about studies with human participants or human data. See also policy information about sex, gender (identity/presentation), and sexual orientation and race, ethnicity and racism.

Reporting on sex and gender	Inspiration4 mission consisted 4 crew members, 2 males and 2 females. We collected samples from all members from the mission and did not perform any gender-specific analysis.
Reporting on race, ethnicity, or other socially relevant groupings	Inspiration4 mission consisted 4 crew members and we collected samples from all members from the mission. We did not selected and performed analysis for specific race, ethnicity, or other socially relevant groupings because of small sample size.
Population characteristics	See above
Recruitment	All four members from the mission consented to share samples for research and we believe the selection bias is minimized as we recruited all members from the mission.
Ethics oversight	The procedure followed guidelines set by Health Insurance Portability and Accountability Act (HIPAA) and operated under Institutional Review Board (IRB) approved protocols. Experiments were conducted in accordance with local regulations and with the approval of the IRB at the Weill Cornell Medicine (IRB #21-05023569).

Note that full information on the approval of the study protocol must also be provided in the manuscript.

Field-specific reporting

Please select the one below that is the best fit for your research. If you are not sure, read the appropriate sections before making your selection.

☒ Life sciences ☐ Behavioural & social sciences ☐ Ecological, evolutionary & environmental sciences

For a reference copy of the document with all sections, see nature.com/documents/nr-reporting-summary-flat.pdf

Life sciences study design

All studies must disclose on these points even when the disclosure is negative.

Sample size	95 ROIs across 2 time-points, 4 study subjects. Inspiration4 mission had 4 crew members, and biopsies were allowed only before and after spaceflight (due to logistics and clinician availability).
Data exclusions	No data were excluded - all the samples were sequenced/analyzed
Replication	Both technical and biological replicates were considered in the analysis. Statistical tests used in the analysis were explained in the figures and legends. To validate the findings, RNAscope and IHC experiments were performed for selected set of markers. Immune-related changes were compared with cytokine and multiome assays, and microbial/epithelial layer changes were compared with metagenomics/ metatranscriptomics assays.
Randomization	Timepoints (pre- and post- spaceflight) and skin compartment types were grouped during analyses and all the samples were treated as replicates, therefore randomization was not relevant.
Blinding	For most of the analyses, individual-level information was not used in the study. Therefore, blinding was not relevant for most of the analysis.

Reporting for specific materials, systems and methods

We require information from authors about some types of materials, experimental systems and methods used in many studies. Here, indicate whether each material, system or method listed is relevant to your study. If you are not sure if a list item applies to your research, read the appropriate section before selecting a response.

Materials & experimental systems

n/a	Involved in the study
☐	☒ Antibodies
☒	☐ Eukaryotic cell lines
☒	☐ Palaeontology and archaeology
☒	☐ Animals and other organisms
☒	☐ Clinical data
☒	☐ Dual use research of concern
☒	☐ Plants

Methods

n/a	Involved in the study
☒	☐ ChIP-seq
☒	☐ Flow cytometry
☒	☐ MRI-based neuroimaging

Antibodies

Antibodies used	Immunofluorescent visualization marker for Pan-Cytokeratin (PanCK, Novus cat# NBP2-33200AF532, Alexa Fluor® 532, clone ID AE1 + AE3, 1:40 dilution), fibroblast activation protein (FAP, Abcam cat# ab222924, clone ID EPR20021, conjugated to Alexa Fluor® 594 using ThermoFisher antibody labeling kit cat# A20185, 1:20 dilution) and smooth muscle actin (SMA, R&D Systems cat# IC1420R, Alexa Fluor® 647, clone 1A4, 1:200 dilution) were used for region or interest (ROI) selection
Validation	Antibodies described above were used for human tissue immunofluorescence staining.
